# Experiential History as a Tuning Parameter for Attention

**DOI:** 10.5334/joc.25

**Published:** 2018-05-14

**Authors:** J. H. Kryklywy, R. M. Todd

**Affiliations:** 1 Department of Psychology, University of British Columbia, CA; 2 Djavad Mowafaghian Centre for Brain Health, University of British Columbia, CA

**Keywords:** Attention, Emotion and cognition, Implicit cognition

## Abstract

[Peer commentary on “Visual selection: usually fast and automatic; seldom slow and volitional,” by J. Theeuwes]. *Journal of Cognition*.

In his current opinion piece, Theeuwes emphasizes the role of selection history as a third source of attentional selection, beyond top-down and bottom-up mechanisms, thus challenging traditional dual-process models of attention. While we agree that selection history impacts the allocation of attention, our own work suggests that this terminology may be too restrictive, and propose the simple term *history* as a better reflection of the impact of learning on our selection biases. Furthermore, we propose that the role of selection/experiential history on attention may not be as a unique third source of attentional selection, but rather as a tuning parameter, allowing certain categories of item to be endowed with greater task-based or feature-driven salience in a context and history dependent manner. This conceptualization presents an alternative to abandoning dual-process models of attention altogether. Rather, we can reimagine how task-based and feature-driven processes may be controlled by past experience in a dynamic and adaptable system.

## Commentary

In his current opinion paper Theeuwes (2018) reemphasizes the addition of selection history as a third source of attentional selection, alongside traditionally-conceived task-based and feature based sources, thus continuing to challenge dual process frameworks that dichotomize attention into top-down and bottom up processes. To support his position, he reviews evidence from priming, fear and reward conditioning, and statistical learning. Furthermore, he emphasizes the importance of experience in influencing the plasticity of visual priority maps and the implicit changes in the representation of a feature or location within the map.

For our part, we have built on this framework, initially proposed by Awh et al., (2012), by emphasizing longer-term temporal processes and the effects of developmental experience on attentional selection (Markovic, Anderson, & Todd, 2014; Todd & Manaligod, 2017). Our own research has found that traumatic experience, both in the presence of PTSD and in healthy participants, tunes attentional prioritization to trauma-related stimuli for years after the traumatic experience (Lee, Todd, Gardhouse, Levine, & Anderson, 2013; Todd et al., 2015). We have also found that genetically-conferred individual differences in neuromodulator availability can tune attentional biases to emotionally salient aspects of the environment, potentially through their influence on emotional learning over the course of development (Ehlers & Todd, 2017; Todd et al., 2013). Beyond long-term effects of emotional learning — which may reflect real-world experiences equivalent to aversive and appetitive conditioning implemented in laboratory settings — we have observed that political affiliation and reported levels of concern about climate change prioritize attention to climate-related cues, even in the absence of priming or explicit attention to those cues (Whitman, Zhao, Roberts, & Todd, 2018). Finally we have found that simply assigning ownership to images of objects is enough to reliably induce prioritization, measured in a temporal order judgment task, raising interesting questions about the relationship between self, value, and attention (Truong, Roberts, & Todd, 2017). Together these findings suggest that, outside of the lab, long-term life history plays a crucial role in tuning attention in numerous complex ways. All of these may influence the representation of features or objects within a priority map. Reflecting our own emphasis on long-term processes, we have suggested it would be more accurate if the myriad influences of learning on selection bias be simply referred to as *history*.

In addition to focusing on a developmental time-scale, we have also emphasized the dynamic nature of the priority landscape, with constant shifts in topography that accompany changes in what is currently relevant (Todd & Manaligod, 2017). These are in turn informed by memory — by neurocognitive patterns developed through experience activated in a manner specific to a particular context and the organism’s perceived state of the world. For example, the aspects of the same winter landscape that capture attention for their threatening or rewarding qualities will be different when driving on slippery roads from when we are snowshoeing, and different again for one who grew up with the harsh Canadian winters compared with the warm Mediterranean sun. For this reason we have proposed that it is useful to think about a priority *state-space*, or a dynamic landscape subject to phase transitions in which the map’s topography may be rapidly and radically rearranged in a context-specific manner.

While our previous taxonomy stressed an increasing number of processes implicated in the influence of “history” on attentional selection, one worry with this ever-expanding source of salience is that the observation that everything is included renders the taxonomy a meaningless catch-all. But if we are thinking of the attentional landscape in terms of priority state spaces, maybe there is another way to integrate the influence of history on attention. Perhaps, rather than being a third source of salience, experiential history and learning act to tune the system’s parameters, such that in any given state of the world, certain categories of items will be endowed with greater task-based or feature-driven salience. As we think of the attentional landscape as a dynamic state space, we can think of historical factors as potential tuning parameters for this space; they are the factors that determine what will attract and repel attention. In this case, with a change of situation, historical factors can tip the landscape into a new state where new top-down goals and low-level features lose or acquire salience in context-specific ways. For example, there is evidence that emotional salience, resulting from associative learning processes, interacts with visual salience to shape attentional priorities (Mather & Sutherland, 2011; Vieira, Wen, Oliver, & Mitchell, 2017). Thus, the same given set of physical inputs results in dramatically different attentional landscapes. Moreover, the notion of feature-driven attention, while often conceptualized as purely stimulus-dependent, inherently depends on the ability of a given system to detect these salient features. Our visual system may have an evolutionary predisposition to give specific stimulus features (e.g., colour, shape etc.) preferential access to attentional resources; however, through experiential learning, we also have the ability to reorganize the underlying physical systems to exhibit greater reactivity to formerly neglected features while reducing the previously afforded sensitivity to others. History can thus result in an ever-changing set of factors capable of engaging feature-based prioritization. Moreover, it is not just feature-based attention that is tuned by history as outside of the lab— experience can be seen to consistently shape our task-based goals as well.

Ultimately, if we think of this broad category of history as a parameter that dynamically tunes the landscape, rather than a third source of salience guiding attentional selection, perhaps a dual-process model of ever-changing task and feature-based salience may be the most parsimonious model after all.

## Data Accessibility Statement

The authors have no data accessibility statement to declare.
